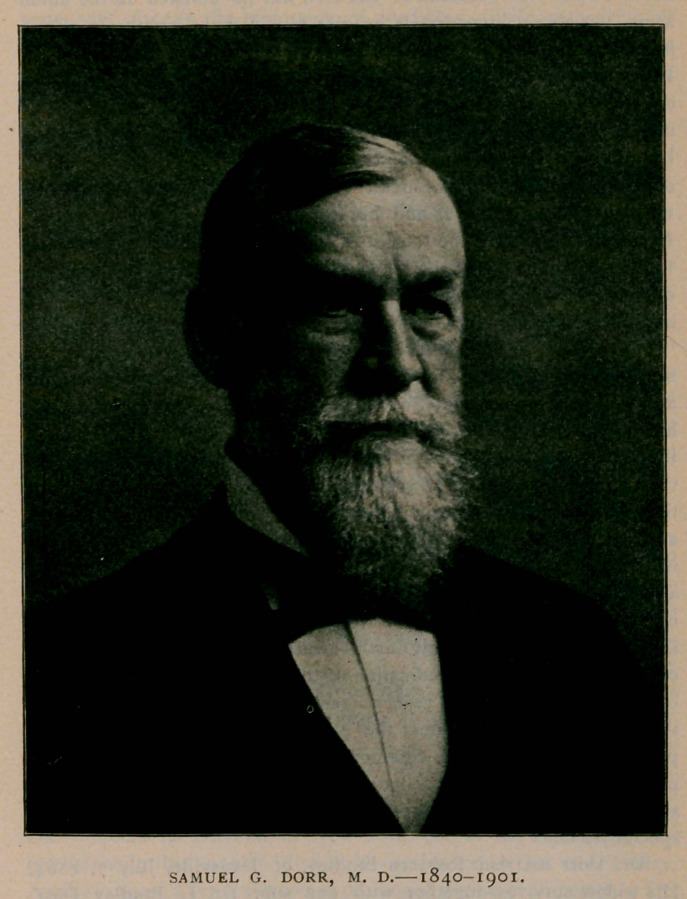# Death of Dr. Samuel G. Dorr

**Published:** 1901-05

**Authors:** 


### OBITUARY.

### DEATH OF DR. SAMUEL G. DORR.

Dr. Samuel Griswold Dorr, U. of B., '75, postmaster of Buffalo, died of angina pectoris, at his home in this city, Sunday morning, April 28, 1901, aged 61. He left the postoffice at 7 o'clock the evening previous, apparently in his usual good health. He retired early without complaint and slept well all night; but about 6 o'clock Sunday morning he called his son, Dr. L. Bradley Dorr, an accomplished physician, on account of severe pain in his left chest. Dr. Dorr, Jr., recognising the severity of the attack, began at once to administer the usual remedies; but they did not avail except in the most temporary way, and his father expired near 8 a. m., or only about two hours after the known onset of the malady. The suddenness of the death came as a great shock to the entire city. Dr. Dorr came of ancient German lineage and Edmund Dorr, one of his ancestors, came from England to Connecticut early in the XVIII. century. Captain Matthew Dorr, another of his ancestors, distinguished himself in the war of the revolution. Edmund Dorr,

SAMUEL G. DORR, M. D.—1840–1901.

before alluded to, married a member of the distinguished Griswold family, from which Dr. Dorr derived his middle name.

Samuel Griswold Dorr was born at Dansville, N. Y., May 30, 1840, where he spent a portion of his early life. He received his preliminary education at Nunda Academy in this state and at the Albion State Academy in Wisconsin. After graduating from the latter he returned to Dansville, where he engaged in the milling business. Upon the outbreak of the civil war he enlisted in the union army, but almost immediately he was seized with diphtheria, which invalided him for a year, hence he was unable to go to the field. In 1863, however, he was appointed a recruiting agent in Livingston county, where he rendered valuable service during the remainder of the war period. After peace came he engaged in the oil refining business in Pennsylvania and established a barrel factory as a collateral branch of the business. These occupations, however, were not suited to his desires and he turned his attention to medicine, following in this way the traditions of his family. In 1873 he came to Buffalo and entered the University as a student. He took his doctorate degree with honor in 1875 and immediately established himself in practice in this city.

Dr. Dorr soon found himself busily engaged in his profession, which he continued to practise until his death. His clientèle was numerous, attached, even affectionate, and he possessed the respect, not only of his colleagues in the medical profession, but of a very large community that knew him well, and with whose kindly acts and gentle demeanor they were familiar. He served two terms as police surgeon, was at one time a member of the consulting staff of the Sisters of Charity Hospital and a member of the several local medical societies. In 1888 he served in the national republican convention at Chicago. In 1899 he was appointed postmaster of Buffalo by President McKinley and entered upon the duties of that office April 1, in that year. He faithfully served in that capacity until death so ruthlessly terminated his official career. At the recent dedication of the new post office building his courtly figure will be well remembered by the thousands present on that occasion, as he sat near the postmaster general and modestly performed his part in the ceremonies. During the progress of the proceedings he dictated a letter to the President that was sealed and posted by the postmaster-general and sent to Washington in a special pouch.

Dr. Dorr married Rebecca Bradley, of Dansville, July 7, 1864. His widow survives, together with one son, Dr. L. Bradley Dorr, and five daughters. His funeral was largely attended on Tuesday, April 30, by physicians and citizens in general, many of whom were intimate personal friends of the distinguished physician. The remains were intered in the family lot at Dansville, N.Y.

## Figures and Tables

**Figure f1:**